# Cost Drivers and Financial Burden for Cancer-Affected Families in China: A Systematic Review

**DOI:** 10.3390/curroncol30080555

**Published:** 2023-08-16

**Authors:** Yufei Jia, Weixi Jiang, Bolu Yang, Shenglan Tang, Qian Long

**Affiliations:** 1 Global Health Research Center, Duke Kunshan University, Kunshan 215316, China; yufei.jia@dukekunshan.edu.cn (Y.J.); bolu.yang@duke.edu (B.Y.); shenglan.tang@duke.edu (S.T.); 2 School of Public Health, Fudan University, Shanghai 200032, China; jiang_weixi@fudan.edu.cn; 3 Duke Global Health Institute, Duke University, Durham, NC 27708, USA; 4 SingHealth-Duke-NUS Global Health Institute, National University of Singapore, Singapore 119007, Singapore

**Keywords:** cancer, cost, financial burden, coping strategies

## Abstract

This systematic review examined cancer care costs, the financial burden for patients, and their economic coping strategies in mainland China. We included 38 quantitative studies that reported out-of-pocket payment for cancer care and patients’ coping strategies in English or Chinese (PROSPERO: CRD42021273989). We searched PubMed, Embase, Ovid, Web of Science, Cochrane, CNKI, and Wanfang Data from 1 January 2009 to 10 August 2022. We referred to the standards for reporting observational studies to assess the methodological quality and transparent reporting of the included studies and reported the costs narratively. Annual mean medical costs (including inpatient and outpatient costs and fees for self-purchasing drugs) ranged from USD 7421 to USD 10,297 per patient. One study investigated medical costs for 5 years and indicated that inpatient costs accounted for 51.6% of the total medical costs, followed by self-purchasing drugs (43.9%). Annual medical costs as a percentage of annual household income ranged from 36.0% to 63.1% with a metaproportion of 51.0%. The common coping strategies included borrowing money and reduction of household expenses and expenses from basic health services. Costs of inpatient care and self-purchasing drugs are major drivers of medical costs for cancer care, and many affected households shoulder a very heavy financial burden.

## 1. Introduction

In 2020, cancer was the leading cause of death in China with a total of 3 million deaths. This accounted for 30% of all cancer deaths in the world [1]. With the demographic and epidemiological transition in China, the continued increase in new cancer cases not only imposes burdens on individuals and the health system, but also leads to productivity losses for society due to premature mortality from cancer [2,3,4]. It is estimated that the total cost of lost productivity attributable to cancer-related premature death in China in 2012 was USD 28 billion, corresponding to 0.34% of the total gross domestic product (GDP) [3].

In China, cancer survival has remarkably increased over the past two decades; however, geographic, social, and economic inequalities in cancer outcomes remain [2,5,6]. The socially and economically disadvantaged population is more likely to delay access to cancer care and subsequently have inadequate treatment, which is often associated with poorer cancer prognosis [2,6]. The most common hindrance to cancer treatment is financial hardship, which includes expensive medical costs, high nonmedical costs for access to care, and loss of income. Families with cancer patients often experience heavy financial burdens caused by cancer care; moreover, some families are driven into poverty [7]. Financial issues are also a prevalent source of distress among cancer patients, and the presence of cancer-related financial distress has been linked to an increased risk of adverse psychological and emotional outcomes, as well as a diminished quality of life [8,9,10,11].

The international community has acknowledged that achieving universal health coverage (UHC), equal access to high-quality healthcare without suffering from financial hardship, is essential to move towards other health-related Sustainable Development Goals (SDGs), including a one-third reduction in premature mortality from noncommunicable diseases by 2030 [2]. In 2009, China launched an in-depth healthcare system reform with a rapid scaling up of essential health insurance coverage (including Urban Employee Basic Medical Insurance (UEBMI), Urban Resident Basic Medical Insurance (URBMI), and New Cooperative Medical Scheme (NCMS) for rural residents), which aimed to protect patients with severe diseases from financial risks [12]. When the health insurance schemes were initiated, they primarily provided a relatively high reimbursement proportion for inpatient care but very limited coverage for outpatient care. In 2013, eight cancers were covered by the catastrophic insurance for rural residents, indicating no less than 70% of reimbursement for inpatient care [13]. In 2015, the Chinese government launched the first round of negotiations on drug procurement, including a couple of first-line anticancer drugs [14]. Since then, more drugs for cancer treatment have been covered by essential health insurance schemes. By 2020, a total of 52 drugs for several common cancers in China were included [15,16]. In addition, the urban and rural resident health insurance schemes increased the coverage for outpatient care for expensive chronic diseases (including cancer) in 2019 [17].

Despite the efforts China has made to provide financial protection for cancer patients, evidence is limited about how affected families afford cancer care. We conducted a systematic review of medical costs, direct nonmedical costs, and indirect costs incurred by cancer care as well as the financial burden placed on families with cancer patients and their economic coping strategies. Our aim was to provide evidence for the scale-up of social protection strategies to mitigate risks of financial hardship caused by cancer.

## 2. Material and Methods

### 2.1. Eligibility Criteria and Search Strategy

The protocol of the systematic review was registered in PROSPERO (registration number: CRD42021273989). In this systematic review, we included original quantitative studies conducted in mainland China that reported direct medical costs, nonmedical costs, indirect costs for cancer care paid out of pocket, and patients’ coping strategies. We screened articles in English and Chinese and only included those published between January 1 January 2009 and 10 August 2022. Quantitative data from longitudinal and cross-sectional studies and baseline data from experimental studies were eligible for inclusion. We used data from 2009 onwards for analysis in correspondence to the new round of health system reform in China. Studies that used secondary data derived from other published articles, did not explicitly report on the methods used for data collection or data analysis, or did not report on patient costs were excluded.

We searched the following databases: PubMed, Embase, Ovid, Web of Science, Cochrane, and two Chinese databases (CNKI and Wanfang Data) with a combination of the key search terms “cancer, tumor, carcinoma, oncology; cost, expenditure, payment, spending, financial, burden; insurance, social protection; China, Chinese”. We presented the search strategy for each database in Table S1. In addition to the database search, we screened the reference lists of included studies to identify relevant studies. We used Covidence to manage all citations and removed duplicates, which facilitated the review of all articles by two independent reviewers. First, YJ, WJ, BY, and two trained undergraduate students were paired and independently screened titles and abstracts. Then, YJ, WJ, and BY retrieved the full texts for evaluation if a citation was considered relevant or uncertain for inclusion or exclusion due to insufficient information obtained from the title and abstract, extracted data from the included studies, and conducted quality assessments of included studies. A third reviewer (QL) was involved in resolving discrepancies and uncertainties at the selection stage, data extraction, and quality assessment.

### 2.2. Data Extraction and Quality Assessment of Included Studies

We developed a data extraction form including the following information: study settings, type of cancer, study design, study period, data source, and methods of data collection, as well as outcomes (including components of costs, financial burden, and coping strategies). We extracted data on costs by medical costs (with stratification of inpatient and outpatient costs), nonmedical costs, and indirect costs according to available data from the included studies. When possible, we also extracted cost data by patients’ demographic (e.g., age, sex), socioeconomic status (e.g., type of health insurance), and clinical stage of cancer. Additionally, we extracted data on the percentage of households with a cancer patient facing catastrophic expenditure, defined by the study authors, as well as coping strategies where available. One reviewer extracted the data, and another reviewer checked them. A third reviewer assessed and reached a consensus on data extraction. If the information was unclear or lacking details, we contacted the authors for clarification and additional information.

According to the standards for reporting observational studies (STROBE, NEWCASTLE, and Circum Network’s Quality Criteria for Survey Research), we introduced ten quality criteria to assess the methodological quality and transparent reporting of quantitative studies (Table S2), which were included in this review [18,19,20]. Each criterion was scored with one point if the assessment was satisfactory. The score for each study could be ranging from 0 to 10. We valued the overall quality of a study as ‘low’, ‘middle’, or ‘high’ if the score was lower than, equal to, or higher than the median score across all included studies, respectively. One reviewer conducted the quality assessment of all included studies, and a second reviewer checked and discussed with a third reviewer if there was any discrepancy.

### 2.3. Data Analysis

Given the large heterogeneity across studies, we reported the distribution by components of costs during the study periods defined in each study. We recorded the range of means across studies and calculated the unweighted average of means (with standard deviation) and the median of mean costs (with an interquartile range of means) for all cost components. We summarized the subgroup costs by age, sex, type of health insurance, and stages of cancer when data on cost components were available. If one study reported data for several different types of cancers, each type of cancer was involved in the analysis as an individual observation. All data on costs were converted from Chinese yuan to US dollar based on the exchange rate in 2020 [21]. We adjusted for inflation using the consumer price index (CPI) for healthcare on medical costs and the general CPI on nonmedical costs, indirect costs, and annual household income [22].

We also presented the range of percentages accounting for different cost components in the cost breakdown analysis. Furthermore, we calculated the annual out-of-pocket payment for cancer care as a percentage of annual household income and conducted a meta-analysis of proportions using STATA 16 [23]. In addition, we presented the range of percentages of catastrophic health expenditure and coping strategies reported in the studies narratively.

## 3. Results

### 3.1. Study Identification and Main Characteristics of the Studies

**Figure 1:**
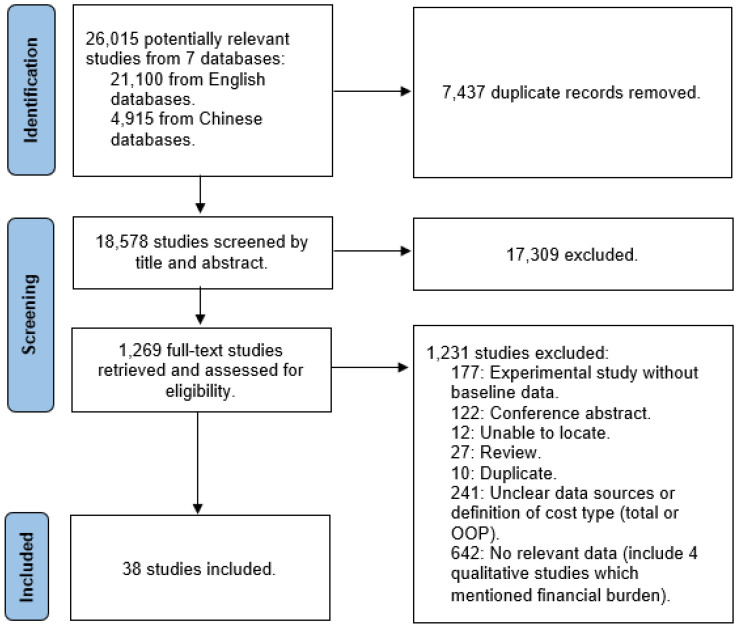
PRISMA flow diagram.

Figure 1 presents the search, screening, and study inclusion and exclusion profile. After screening titles and abstracts, we retrieved 1269 studies for full-text assessment. Of these studies, we excluded 1231 studies largely due to no relevant data, unclear data sources, and ambiguous cost components reported in the study. A total of 38 studies (23 in English and 15 in Chinese) were included.

We presented the main characteristics of the included studies in Table 1. All studies were cross-sectional studies, and data for the studies were obtained from patient surveys (or surveys in combination with data extraction from hospital records or health insurance records) (*n* = 25), hospital-based payment records (*n* = 4), and health insurance records (*n* = 9). A total of 25 studies reported annual mean costs of cancer treatment [24,25,26,27,28,29,30,31,32,33,34,35,36,37,38,39,40,41,42,43,44,45,46,47,48], 1 study reported the average monthly medical cost [49], 1 study reported the average cost of cancer care for 5 years [50], and 3 studies reported the costs during the illness without specifying the course of treatment [51,52,53]. Four studies only reported the median of the costs, which were not included in the data synthesis [54,55,56,57]. In addition, 2 studies only reported coping strategies [58,59], and 2 reported the proportion of catastrophic expenditure without reporting cost data [60,61]. According to the quality assessment for all included studies, 10 studies [30,41,42,43,44,45,51,55,58,61] were assessed as high quality, 10 studies [31,32,33,40,46,47,50,56,57,60] at the middle level, and 18 studies [25,27,28,34,35,36,37,38,39,48,49,52,53,59] as low quality.

### 3.2. Distribution of Cost Components

#### 3.2.1. Medical Costs

This section reported total medical costs and inpatient and outpatient costs across studies (Table 1). A total of 15 studies (including 21 observations, more than one type of cancer in a study) reported annual medical costs for cancer treatment (Table S3) [24,26,31,32,33,35,36,38,40,41,42,43,44,45,46]. Of these, the mean medical costs including inpatient and outpatient costs across 12 studies (18 observations) ranged from USD 1802 to USD 14,684 with an unweighted average of USD 4530 and a median of USD 3947 [26,31,32,33,35,36,38,40,42,43,44,46]. The other three studies included medical costs of inpatient and outpatient care and self-purchasing drugs from pharmacies, and the average costs ranged from USD 7421 to USD 10,297 with an unweighted average of USD 8794 [24,41,45].

The mean annual inpatient costs from 12 studies (including 33 observations) ranged from USD 1369 to USD 8647 with an unweighted average of USD 3146 and a median of USD 2843 (Table S3) [25,26,27,28,29,30,34,37,38,39,44,47]. The average outpatient costs in a year from 5 studies (including 5 observations) varied from USD 115 to USD 865 with an unweighted average of USD 626 and a median of USD 772 [26,29,34,38,44]. Of the two studies that reported both inpatient and outpatient costs per patient incurred within a year, the inpatient costs accounted for 82.0% and 94.3% of the total medical cost, respectively [26,44].

**Figure 2:**
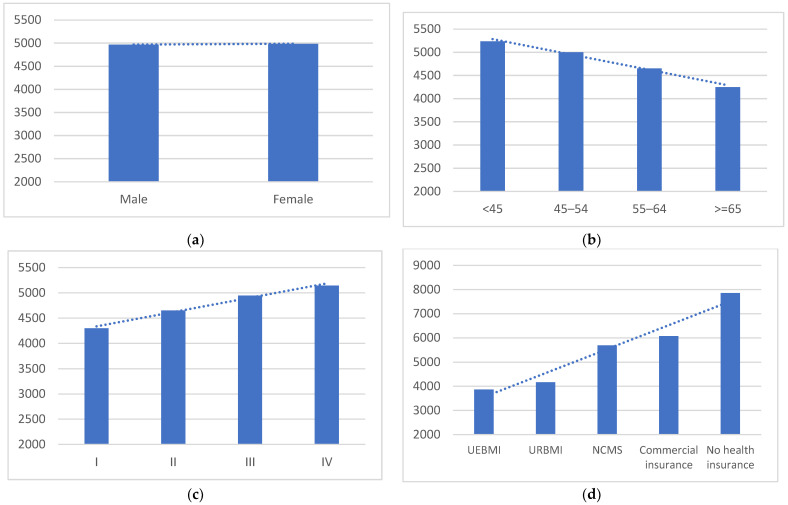
Unweighted average annual medical costs by subgroups from a multicenter patient survey (four studies [31,33,40,42]), USD. (**a**) Sex; (**b**) age groups; (**c**) clinical stage; (**d**) insurance type; (**d**) UEBMI: Urban Employee Basic Medical Insurance; URBMI: Urban Resident Basic Medical Insurance; NCMS: New Cooperative Medical Scheme. See details of the annual medical cost by subgroup reported by these four studies in Table S4.

Four studies from a multicenter patient survey reported medical costs (both inpatient and outpatient costs) for 12 months (2 months before and 10 months after diagnosis) stratified by age, sex, stages of cancer, and type of health insurance for different types of cancer [31,33,40,42]. The unweighted annual medical costs by subgroups are shown in Figure 2. Patients over 65 years old often spent less on cancer care, while there was no significant difference in annual medical costs by sex. In terms of the stages of cancer, patients at stage IV often spent more on cancer care than patients at stage I. In a stratification of health insurance type, cancer patients with UEBMI paid the least medical cost out of pocket within a year, followed by patients with URRBMI, and patients with NCMS and commercial health insurance paid the most. In these studies, few patients did not have any type of health insurance, and the average annual medical costs ranged from USD 6900 to USD 8702 with an unweighted average of USD 7857 and a median of USD 7913. In addition, one patient survey reported inpatient and outpatient costs and costs of self-purchasing drugs from pharmacies within a year, and the mean cost among patients covered by UEBMI was USD 7984 compared with the average cost of USD 9441 among patients covered by URBMI [45]. Another study based on health insurance records reported that the average annual out-of-pocket payments by patients with UEBMI and URBMI were USD 1741 and USD 2507, respectively, which were lower than the data obtained from patient surveys [44].

In addition, 1 study reported that the medical cost for 5 years among 195 surviving lung cancer patients was USD 36,034 on average [50]. Of that, inpatient costs (USD 18,590) accounted for 51.6% of the total medical costs, followed by purchasing drugs from pharmacies (43.9%, USD15,812) and outpatient costs (4.5%, USD 1632).

#### 3.2.2. Nonmedical Costs

Items for nonmedical costs varied across studies but largely included costs on transportation and lodging for both patients and family members as well as fees for extra nutritional products for patients and hiring care workers (Table S5). The average annual nonmedical costs from eight studies (eight observations) ranged from USD 688 to USD 4978 with an unweighted average of USD 1486 [24,29,31,33,40,41,42,43].

#### 3.2.3. Direct Costs

Across seven studies that reported both annual medical and nonmedical costs, the average direct costs for cancer care within a year varied from USD 4903 to USD 12,399 (Table S6) [24,31,33,40,41,42,43]. Of that, the proportion of medical costs accounting for total direct costs ranged from 59.9% to 88.1%. One study reported medical costs and nonmedical costs for 5 years of the treatment, and nonmedical costs accounted for 6% of the total direct cost (USD 38,313) [50].

#### 3.2.4. Indirect Costs

The estimation of indirect costs varied across seven studies without a clear definition in one study (Table S7) [24,30,43,48,50,51,53]. We estimated the percentage of the indirect cost out of the overall costs (total of direct and indirect costs) from four studies based on available data [24,43,50,53]. One study estimated annual indirect costs including loss of income for both lung cancer patients and caregivers and financial loss due to seeking nonmedical support for the illness, which was USD 9978 on average accounting for 44.6% of annual overall costs [24]. Another study only accounted for time loss of both esophageal cancer patients and caregivers caused by cancer treatment and estimated mean USD 1086 annual indirect costs, around 17.4% of annual overall costs [43]. One study investigated parents’ time loss due to taking care of children with retinoblastoma (*n* = 50) during a treatment lasting 23–58 months, which accounted for 32.6% of overall costs [53]. The last study estimated time loss for both lung cancer patients and caregivers due to hospital visits within 5 years of the treatment and reported the lowest indirect costs, on average USD 959 [50].

### 3.3. Financial Burden on Households with Cancer Patient and the Coping Strategies

We calculated the annual medical costs as a percentage of annual household income across eight studies where data were available (Table 2) [24,31,32,33,40,42,45,46]. This proportion ranged from 36.0% to 63.1% with a metaproportion of 51.0%. Across five studies that reported both annual medical and nonmedical costs, annual direct costs as a percentage of annual household income ranged from 43.6% to 91.5% with a metaproportion of 64.0% (Table S8) [24,31,33,40,42]. One study investigated the financial burden of patients with esophageal cancer (*n* = 184) from a cancer specialty hospital in central China and found that the average annual medical cost was 1.3 times a patients’ annual household income [43].

Nine studies reported the rates of catastrophic health expenditure (CHE) incurred by cancer care (Table S9) [30,41,45,46,47,49,54,58,59]. Five studies defined CHE as an annual medical cost accounting for more than 40% of a household’s nonfood expenditures [41,45,46,47,59]. Of five studies, one study that used nationally representative data among adults over 45 years old obtained from the 2011 and 2015 China Health and Retirement Longitudinal Studies estimated the rates of CHE to be 25.1% in 2011 and 27.2% in 2015 for all types of cancer patients; however, this study found that only half of the participants had received cancer treatment, which may underestimate the CHE rates given those who gave up treatment due to financial difficulties. This study also highlighted patients’ socioeconomic and urban–rural status associated with the utilization of cancer care. The other four studies recruited cancer patients who accepted the treatment and reported rates of CHE ranging from 43.4% to 78.1%. In addition, one study estimated the financial burden placed on cancer patients caused by inpatient care in a year [30]. In this study, 20.6% of cancer patients paid for inpatient treatment, which was more than 40% of annual household income. Two studies defined CHE as annual direct costs (including both medical and nonmedical costs) over 40% of annual households’ nonfood expenditure or income, in which the CHE rates ranged from 49.6% to 72.7% [54,58]. One study investigated the CHE rate during the last 3 months of life among advanced cancer patients and found that more than 90% of households (94.3% of urban households and 96.1% of rural households) spent over 40% of their monthly income on care [49].

Six studies investigated coping strategies when households with cancer patients shouldered expensive expenditures on cancer care (Table 3) [41,43,48,49,60,61]. All studies reported borrowing money from relatives and friends or a loan. In two more recent studies, the reduction of household expenses (66.2%) and the reduction of expenses on basic health services (41.2%) were common strategies for addressing challenges caused by cancer care [60,61]. One study recruited parents with children diagnosed with leukemia and found that the most common strategy was borrowing money from relatives and friends (97.9%), followed by charity assistance (54.6%) and government subsidy (16.1%) [41].

## 4. Discussion

This systematic review mapped the medical, nonmedical, and indirect costs incurred by cancer care in China from 2009 onwards. This review demonstrated that costs of inpatient care and patients self-purchasing drugs were major components of medical costs. Despite a vast majority of patients being enrolled in different types of health insurance schemes, the out-of-pocket payment for cancer care remained high. Given nonmedical costs and indirect costs incurred by cancer care, the financial burden placed on the affected households was often very heavy, and many families faced catastrophic health expenditures.

### 4.1. Interpretations

Over the past two decades, cancer survival has remarkably increased in China, suggesting improved accessibility and availability in the quality of cancer care [5]. Similar to other low- and middle-income countries, however, the financial risk protection for access to cancer care remains insufficient [2]. In this review, inpatient care was a major component of medical costs. Despite the substantial government investment that almost achieved universal health insurance coverage, the deductible and copayment, particularly at specialty hospitals, are often high [62]. Moreover, many new cancer diagnosis technologies, innovative therapies, and health products are costly yet are not covered by essential health insurance schemes [63]. Over the past decade, the Chinese government continues to increase funding for URBMI and rural NCMS, and most of the provinces have integrated the two schemes into one Urban and Rural Resident Basic Medical Insurance for expanding the risk pools; however, the benefit packages of these two schemes are still more constrained compared with the UEBMI coverage [64]. In alignment with the ongoing health system reform, the government encourages cooperation between public and private health sectors, including pilots of commercial health insurance development serving as a supplementary health insurance scheme of the basic health insurance schemes [62]. It is not surprising to find in this review that patients with UEBMI paid the least medical cost out of pocket compared with patients with other types of health insurance.

Patients self-purchasing drugs was another primary driver of medical cost and was often not reimbursable by health insurance schemes. One study identified that self-purchasing drugs accounted for more than 40% of medical costs within 5 years [50]. Additionally, the annual medical costs, in several studies that counted patients purchasing drugs themselves, were also significantly higher than the average annual medical cost in studies that only investigated costs on inpatient and outpatient care [24,31,33,40,45]. Nevertheless, these studies did not describe the type of drugs purchased by patients themselves or interpretations on motivations or reasons for this patient’s behavior. Other cancer studies in China indicated unaffordable or unavailable patented anticancer medicines or the application of traditional Chinese medicine as a supplementary approach, which may partially explain the costs incurred by patients purchasing drugs themselves [2,65,66].

In this review, although the annual medical costs varied by type of cancer, the costs of late-stage cancer were often higher than the costs of early-stage cancer. In 2005, China initiated the cancer screening and early detection program (including female breast and cervical cancers, liver, stomach, esophageal, and nasopharynx cancers) and gradually expanded to all provinces in mainland China by 2015 [67]. The implementation and the effects of the cancer screening program between urban and rural areas and across regions in China should be further evaluated. Awareness of and willingness to have active medical examinations may contribute to early access to cancer care and are associated with less expensive treatments at the early stage.

This review found that the estimation of nonmedical costs and indirect costs varied across studies, and thus, the ranges of nonmedical costs and indirect costs as a percentage of direct cost and overall cost for cancer care were wide. There are insufficient details to allow us to break down the analysis of nonmedical costs for transportation and lodging, additional nutrition products, or hiring a care worker. Indirect costs often refer to time or productivity loss of cancer patients or their family caregivers, but the measure is not consistent across studies. The available data imply the importance of measuring the financial burden considering relevant cost components in order to propose strategies to mitigate financial risks as a result of cancer care.

This review demonstrated that direct costs incurred by cancer care imposed a heavy financial burden on the affected families, and more than half of the households faced catastrophic health expenditures. The most common coping strategy was to borrow money. Meanwhile, the households reduced family expenses and even expenses on basic health services. Elderly cancer patients often paid less for cancer care; they have limited capacity to pay and are afraid of the financial burden extended to their adult children [68]. For childhood cancers, some families received charity assistance or government subsidy; however, the indirect costs due to taking of care ill children were often high [41]. Thus, efforts to provide financial protection for cancer-affected families should consider a wide range of cost impacts.

### 4.2. Policy and Research Implications

Cancer is a major cause of premature death and often exposes people to an economic shock, which disproportionately affects individuals in low- and middle-income countries [69,70]. A global survey led by WHO found that a vast majority of countries developed cancer or NCD prevention and control policies, strategies, or plans [63]. However, knowledge of the effective implementation of national plans remains limited, including practical priorities, resource allocation, a means of evaluation, and others. A global analysis highlights context-specific evidence for cancer control plan development in low- and middle-income countries [71]. We interpreted findings embedded in the comprehensive health system reform in China and proposed relevant policy and research implications, which would be also valuable for other low- and middle-income countries facing similar challenges.

In correspondence with the national health plan “Healthy China 2030”, China’s Cancer Prevention and Treatment Action Plan (2015–2017) highlighted strengthening stewardship for cancer control including multisector cooperation to mitigate the risk of financial hardship [5]. Compared with the 2012 national essential medicine list, eleven new antitumor medicines were included in 2018. In the same year, the first list of generic drugs, a total of 34 drugs, was published [72]. More antitumor medicines are also included in the reimbursement list of essential health insurance schemes through government drug procurement in recent years. In this review, a vast majority of studies were conducted before 2018. The effects of cancer control strategies adopted recently need further monitoring. Our review showed that the financial burden remains high, especially high for studies conducted after 2015 compared with the studies conducted before 2015 or for the same type of cancer [24,45]. This may be partially attributed to a rapid increase in health expenditure on cancer-related new diagnoses and therapies; however, many expensive innovative approaches show only modest benefits for cancer control according to the WHO report on cancer in 2020 [63].

The overview of 10 years of health system reform in China identified the remaining inefficiency in service delivery and uncontrolled escalation of health expenditures [61]. Public hospital financing reform including removing drug markups shows an intended effect of hospital revenue decline from pharmaceutical sales [65,73,74]. There are also unexpected impacts on the increase in expenditure for inpatient and outpatient care and problems with drug access. The major drivers of medical costs for cancer care including costs of inpatient care and patients’ self-purchasing drugs may incite improvement in regulations on cancer clinical services and ensure the security of the medicine and the drug supply to promote value-based cancer care. Additional public and private financial protection schemes and medical assistance (e.g., charity or government subsidies) should be further explored and aim to prevent socioeconomically vulnerable people from financial hardship caused by cancer care. To achieve the targets of cost control and financial risk protection for cancer-affected families, China should strengthen the governance of the health system and the monitoring and evaluation of the related strategies’ implementation.

This review also has implications for future research. First, data on costs, particularly nonmedical, and indirect costs are inconsistent across studies. To gain a better understanding of the components and structures of costs as well as the financial burden of cancer care, a study tool should be standardized and validated in the context. A longitudinal design will be optimal. Second, qualitative or mixed-methods research is needed to gain deeper knowledge on cancer care access, particularly the impact of financial toxicity, to inform interventions supporting vulnerable population. Third, health policy and system research should be conducted to assess related policy implementations and provide evidence-based recommendations to improve quality and equality in cancer control.

### 4.3. Strengths and Limitations

This review generated country-specific evidence on cost drivers and the financial burden of cancer care in the context of the new round of health system reform in China; however, the review had several limitations. First, how outcomes were defined and how data were collected and analyzed, particularly nonmedical and indirect costs, varied considerably across the studies. The estimation and interpretation of the financial burden in this review were mainly based on medical costs. Second, the number of studies that allow us to conduct subgroup analysis by patients’ demographic, socioeconomic status, and type of cancer was limited. We were unable to run a metaregression to examine the association between patients’ characteristics and the financial burden observed. Third, the search strategy was not optimal to identifying studies only reporting the coping strategies used by the cancer-affected households. The literature on coping with the costs of cancer care may not be fully covered. Qualitative studies will be more suitable to deeply exploring common or unintended coping strategies.

## 5. Conclusions

Although China has introduced relevant policies aiming to provide financial protection for access to cancer care in the context of the ongoing health system reform, this review demonstrated that costs of inpatient care and self-purchasing drugs are major drivers of medical costs for cancer care in China, and the financial burden for the affected families remains very heavy. Addressing this burden will require strengthening stewardship for cancer control and multisector cooperation to mitigate the risk of financial hardship. This will also require further in-depth research to gain a better understanding of the individual, social, and health system determinants of the quality of cancer care, in terms of cost containment and financial risk protection, in order to inform cancer control policy development in China.

## Figures and Tables

**Table 1:** Main characteristics of the studies and type of costs.

Study	Type of Cancer	Number of Patients	Study Year	Duration of Costs	Data Source	Mean/Median/Both	Component of Costs			Breakdown of Direct Costs				CHE
										Medical Costs			Non-MD	
							Direct	Indirect	Total	Total	INPT	OPT		
Huang, 2012 ^a,^* [24]	lung cancer	402	2010–2011	annual	survey	mean	√	√	√	√			√	
Xiao, 2012 [25]	multicancer (the elderly)	973	2009	annual	medical record	mean					√			
Peng, 2013 [26]	lung cancer	1216	2009	annual	survey and basic health insurance database	mean				√	√	√		
Jin, 2014 [27]	cancer	5062	2012	annual	basic health insurance (UEBMI) database	mean					√			
Xiao, 2014 [28]	cancer	4854	2010–2012	annual	basic health insurance (NRCMS) database	mean					√			
Li, 2016 ^c,^* [29]	lung cancer	218	2014	annual	survey	mean					√	√	√	
Zhao, 2016 * [30]	multicancer	318	2014	annual	survey	mean		√			√			√
Huang, 2017 * [31]	colorectal cancer	2356	2012–2014	annual ^b^	survey	mean	√			√			√	
Lin, 2017 * [32]	multicancer	252	2015–2016	annual	survey and basic health insurance database	mean				√				
Liao, 2018 * [33]	breast cancer	2746	2012–2014	annual ^b^	survey	mean	√			√			√	
Xie, 2018 [34]	cancer	OPT: 959	2014–2016	annual	basic health insurance (UEBMI) database	mean					√	√		
		INPT: 838												
Du, 2018 [35]	cancer-poor population	207	2012–2016	annual	medical assistance database	mean				√				
Zhang, 2018 [36]	cancer-poor population	122	2017	annual	survey	mean				√				
Yuan, 2019 [37]	colorectal cancer	8021	2012–2016	annual	medical record	mean					√			
Zhuo, 2019 [38]	prostate cancer	1672	2011–2014	annual	basic health insurance (UEBMI) database	both				√	√	√		
Yang, 2019 [39]	multicancer	20,138	2013–2017	annual	medical record	mean					√			
Lei, 2020 * [40]	liver cancer	2223	2012–2014	annual ^b^	survey	mean	√			√			√	
Sui, 2020 ^a,#^ [41]	pediatric leukemia	242	2018	annual	survey	mean	√			√			√	√
Zhang, 2020 * [42]	stomach cancer	2401	2012–2014	annual ^b^	survey	mean	√			√			√	
Chen, 2020 *^,#^ [43]	esophageal cancer	184	2019	annual	survey	mean	√	√	√	√			√	
Zhu, 2021 [44]	lung cancer	38,199	2013–2016	annual	basic health insurance (UEBMI, URBMI) database	mean				√	√	√		
Sun, 2021 ^a,^* [45]	lung cancer	2565	2015–2016	annual ^b^	survey	mean				√				√
Huang, 2021 * [46]	cancer	332	2015–2016	annual ^b^	survey	mean				√				√
Zhao, 2021 * [47]	cancer	2011:53; 2015:111	2011, 2015	annual	CHARLS (population based)	both					√			√
Xiao, 2022 ^#^ [48]	advanced gastroesophageal adenocarcinoma	66	2019	annual	survey	both		√						
Total number of studies				25			7	4	2	15	12	5	8	5
Leng, 2019 *,^#^ [49]	cancer patients at the end of life	792	2013–2016	monthly	survey	mean				√				√
Zhang, 2017 ^a,^* [50]	lung cancer	195	2014	5 years	survey	mean	√	√	√	√	√	√	√	
Ren, 2019 * [51]	children with lymphoblastic leukemia	161	2010	during the first three-phase treatment	survey	both		√					√	
Yaun, 2019 [52]	multicancer	349	2016	during the illness	basic health insurance database	mean				√				
Zhou, 2020 [53]	children with retinoblastoma	50	2015–2017	during the illness	survey and medical records	mean	√	√	√	√			√	
Che, 2016 * [54]	liver cancer	131	2013	annual	survey and medical records	median	√			√	√	√	√	√
Bai, 2020 [55]	prostate cancer	3936	2017	annual	basic health insurance (UEBMI, URBMI) database	median				√				
Zhan, 2022 [56]	children with leukemia	765	2015–2019	during the illness	medical record	median					√			
Zhang, 2022 [57]	liver cancer	8969	2011–2017	annual	basic health insurance database	median				√				
Chen, 2018 [58]	lung cancer	227	2016	monthly	survey	both								√
Sun, 2021 [59]	breast cancer	639	2015–2016	--	survey	--								√
Wang, 2022 ^#^ [60]	colorectal cancer	4428	2020–2021	--	survey	--								
Liu, 2022 ^#^ [61]	breast cancer	627	2021	--	survey	--								
Total number of all included studies				38			10	7	4	22	15	7	12	9

^a^ In these studies, medical costs included inpatient and outpatient costs and fees for purchasing drugs from pharmacies. ^b^ In these studies, costs incurred during 2 months before and 10 months after diagnosis. ^c^ This study reported outpatient costs in the past 6 months and inpatient costs in the past year. * This study reported annual household income. ^#^ This study reported coping strategies. INPT: inpatient, OPT: outpatient, Non-MD: nonmedical, CHE: catastrophic health expenditure.

**Table 2:** Annual medical cost as a percentage of annual household income from nine studies, %.

Study	Participants	Cancer Patients (n)	Year	Data Source	Definition of Medical Cost in the Study	Annual Medical Costs (USD)	Annual Household Income (USD)	Annual OOP Medical Costs/Household Income
Huang, 2012 [24]	lung cancer	402	2010–2011	survey	inpatient, outpatient, and fees for purchasing drugs from pharmacies	7420.85	13,550.73	54.8%
Huang, 2017 ^a^ [31]	colorectal cancer	2356	2012–2014	survey	inpatient, outpatient	5397.75	10,013.99	53.9%
Liao, 2018 [33]	breast cancer	2746	2012–2014	survey	inpatient, outpatient	4105.94	10,990.09	37.4%
Lei, 2020 ^a^ [40]	liver cancer	2223	2012–2014	survey	inpatient, outpatient	4056.78	11,259.70	36.0%
Zhang, 2020 ^a^ [42]	Stomach cancer	2401	2012–2014	survey	inpatient, outpatient	5524.95	9481.50	58.3%
Sun, 2021 ^a^ [45]	lung cancer	2565	2015–2016	survey	inpatient, outpatient, and fees for purchasing drugs from pharmacies	8663.51	13,725.00	63.1%
Lin, 2017 [32]	multicancer	252	2015–2016	survey and basic health insurance database	medical costs related to cancer treatment	2410.07	5265.00	45.8%
Huang, 2021 ^a^ [46]	cancer	332	2015–2016	survey	medical costs related to cancer treatment	10,431.25	16,893.94	61.7%
Chen, 2020 ^b^ [43]	esophageal cancer	184	2019	survey	inpatient, outpatient	4460.12	3454.45	129.1%
Random pooled ES (95%CI) of the proportion of the annual medical cost to annual household income from eight studies								51% (44%, 59%)

^a^ In these studies, costs incurred during 2 months before and 10 months after diagnosis. ^b^ This study was not included in the analysis of metaproportion.

**Table 3:** Coping with costs incurred by cancer care from six studies.

Study	Participants	Cancer Patients	Year	Location	Cooping Strategy
Sui, 2020 [41]	pediatric leukemia	242	2018	Heilongjiang	- Support from relatives and friends: 97.93% - Government subsidy: 16.12% - Charity assistance: 54.55%
Chen, 2020 [43]	esophageal cancer	184	2019	Anhui	Borrowed money from relatives and friends: 45.10%
Xiao, 2022 [48]	advanced gastroesophageal adenocarcinoma	66	2019	multicountry	Financial support from caregivers: 62.1%
Leng, 2019 [49]	cancer patients at the end of life	792	2013–2016	multicenter	Borrowed money from relatives and friends: 32.10%
Wang, 2022 [60]	colorectal cancer	4428	2020–2021	multicenter	- Borrowed from relatives and friends: 25.5% - Borrowed from financial institutions: 1.7% - Reduced spending on leisure activities: 41.15% - Reduced household expenses: 66.2% - Selling properties: 4.7%
Liu, 2022 [61]	breast cancer	627	2021	national	- Borrowed money or acquired a loan due to illness: 21.21% - Reduced spending on leisure activities: 41.15% - Considered quitting treatment: 11.64% - Delayed treatment for more than 7 days: 5.42% - Failed to take medicine as instructed: 6.7% - Failed to attend medical visits as instructed: 3.35% - Reduced spending on basic health services: 41.15% - At least one coping strategy: 48.01%

## Supplementary Materials

The following supporting information can be downloaded at https://www.mdpi.com/article/10.3390/curroncol30080555/s1. The Supplementary Materials include Table S1: Search strategy; Table S2: Ten criteria for quality assessment of the included quantitative studies; Table S3: Patient costs calculated by observations; Table S4: Annual medical costs by subgroups from a multicenter patient survey (four studies), mean USD (*n*); Table S5: Nonmedical costs for cancer treatment from 12 patient surveys; Table S6: Medical and nonmedical costs from seven studies, mean and percentage of total annual direct costs; Table S7: Indirect costs for cancer treatment from seven studies; Table S8: Annual direct cost as a percentage of annual household income from six studies and metaproportion of five studies, %; Table S9: Catastrophic health expenditure (CHE) incurred by cancer care from nine studies. PRISMA checklist and PRISMA abstract checklist.
