# Supplementary material for: Cost Drivers and Financial Burden for Cancer-Affected Families in China: A Systematic Review
Source: Curr Oncol. 2023 Aug 16;30(8):7654–71. doi: 10.3390/curroncol30080555 (PMC10453571; doi:10.3390/curroncol30080555)
Supplement: Supplementary file 1 [file curroncol-30-00555-s001.zip › Supplementary Material 1_Tables.pdf]

Supplementary Tables

Table S1. Search strategy

Table S2. Ten criteria for quality assessment of the included quantitative studies

Table S3. Patient costs calculated by observations

Table S4. Annual medical costs by subgroups from a multi-center patient survey (four studies), mean US$ (n)

Table S5. Non-medical costs for cancer treatment from 12 patient surveys

Table S6. Medical and non-medical costs from seven studies, mean and percentage of total annual direct costs

Table S7. Indirect costs for cancer treatment from seven studies

Table S8. Annual direct cost as a percentage of annual household income from six studies and meta-proportion of five studies, %

Table S9. Catastrophic health expenditure (CHE) incurred by cancer care from nine studies

Table S1. Search strategy

Database	Query	First round search[a]	Updated search[b]
Pubmed	((((((((cancer) OR (tumor)) OR (malignancy)) OR (neoplasm)) OR (carcinoma)) OR (oncology)) OR (Metastatic)) AND ((((((((((cost[Title/Abstract]) OR (expenditure[Title/Abstract])) OR (expense[Title/Abstract])) OR (burden[Title/Abstract])) OR (payment[Title/Abstract])) OR (((cost[MeSH Terms]) OR (expenditure[MeSH Terms])) OR (payment[MeSH Terms]))) OR (spending[Title/Abstract])) OR (spending[MeSH Terms])) OR ((financial[Title/Abstract]) OR (financial[MeSH Terms]))) OR ((insurance) OR ("social protection")))) AND ((China[Title/Abstract]) OR (Chinese[Title/Abstract]))	3686	793
Embase	#1 'cancer'/exp OR 'cancer' OR 'cancer'/exp OR cancer OR 'tumor'/exp OR 'tumor' OR 'tumor'/exp OR tumor OR 'malignancy'/exp OR 'malignancy' OR 'malignancy'/exp OR malignancy OR 'neoplasm'/exp OR 'neoplasm' OR 'neoplasm'/exp OR neoplasm OR 'carcinoma'/exp OR 'carcinoma' OR 'carcinoma'/exp OR carcinoma OR 'oncology'/exp OR 'oncology' OR 'oncology'/exp OR oncology OR 'metastatic' OR metastatic<br>#2 cost:ti,ab,kw OR expenditure:ti,ab,kw OR expense:ti,ab,kw OR burden:ti,ab,kw OR payment:ti,ab,kw OR spending:ti,ab,kw OR 'cost'/exp OR expenditure OR expense OR 'burden'/exp OR payment OR spending OR financial:ti,ab,kw OR 'financial'<br>#3 'insurance'/exp OR 'insurance' OR 'social protection'/exp OR 'social protection'<br>#4 #2 OR #3<br>#5 china:ti,ab,kw OR chinese:ti,ab,kw<br>#6 #1 AND #4 AND #5	5126	999
Ovid	#1 financial.mp. or cancer.mp. or exp Neoplasms/ or tumor.mp. or exp Neoplasms/ or exp Surgical Oncology/ or exp Oncology Service, Hospital/ or oncology.mp. or exp Oncology Nursing/ or exp Psycho-Oncology/ or exp Medical Oncology/ or exp Integrative Oncology/ or exp Radiation Oncology/ or metastatic.mp. or neoplasm.mp. or exp Neoplasms/<br>#2 cost.mp. or expenditure.mp. or expense.mp. or burden.mp. or health care costs/ or health expenditures/ or economics, hospital/ or economics, medical/ or economics, nursing/ or economics, pharmaceutical/ or exp Health Expenditures/ or spending.mp. or exp Health Care Costs/ or exp health care costs/ or exp health expenditures/ or exp economics, hospital/ or exp economics, medical/ or exp economics, nursing/ or exp economics, pharmaceutical/ or exp Health Care Costs/ or payment.mp. or exp Insurance, Health, Reimbursement/ or insurance.mp. or exp Insurance/ or 'social protection'.mp. or Financial Stress/ or financial.mp. or Financial Support/<br>#3 China.mp. or exp China/ or Chinese.mp.<br>#4 #1 and #2 and #3	2814	269
Web of Science	#1 ((((((((((ALL=(cancer*)) OR ALL=(cancer)) OR ALL=(tumor)) OR ALL=(tumor*)) OR ALL=(neurofibroma)) OR ALL=(neoplasm*)) OR ALL=(carcinoma)) OR ALL=(carcinoma*)) OR ALL=(oncology)) OR ALL=(metastatic*)) OR ALL=(metastat*)	6528	833

Database	Query	First round search[a]	Updated search[b]
	#2 ((((((((((((((TI=(cost)) OR TI=(cost*)) OR TI=(expenditure))) OR TI=(expenditure*)) OR TI=(payment)) OR TI=(payment*)) OR TI=(burden)) OR TI=(expense)) OR TI=(spending)) OR TI=(financial)) OR TI=(burden*)) OR TI=(economic)) OR TI=(economic*)) OR TI=(financial)) OR TI=(financial*) OR ((((((((((((((AK=(cost)) OR AK=(cost*)) OR AK=(expenditure))) OR AK=(expenditure*)) OR AK=(payment)) OR AK=(payment*)) OR AK=(burden)) OR AK=(expense)) OR AK=(spending)) OR AK=(financial)) OR AK=(burden*)) OR AK=(economic)) OR AK=(economic*)) OR AK=(financial)) OR AK=(financial*) OR ((((((((((((((KP=(cost)) OR KP=(cost*)) OR KP=(expenditure))) OR KP=(expenditure*)) OR KP=(payment)) OR KP=(payment*)) OR KP=(burden)) OR KP=(expense)) OR KP=(spending)) OR KP=(financial)) OR KP=(burden*)) OR KP=(economic)) OR KP=(economic*)) OR KP=(financial)) OR KP=(financial*)<br>#3 ((#2) OR ((ALL=(insurance)) OR ALL=(insure*)) OR ALL=(insura*)) OR ALL=("social protection")<br>#4 (ALL=(China)) OR ALL=(Chinese)<br>#5 ((#1) AND #3) AND #4		
Cochrane	cancer OR tumor OR malignancy OR neoplasm OR carcinoma OR oncology OR Metastatic in All Text AND cost OR expenditure OR burden OR payment OR spending OR expense in Title Abstract Keyword AND China OR Chinese in Title Abstract Keyword - with Cochrane Library publication date Between Jan 2009 and Oct 2021 (Word variations have been searched)	52	--
CNKI	主题（瘤 + 肿瘤 + 癌 + 癌症 + 白血病）AND 篇名（费用 + 支出 + 负担）	941	130
Wanfang data	主题:(瘤 OR 癌 OR 白血病) and 题名或关键词:(费用 OR 支出 OR 经济负担)	3777	67
Total		22924	3091

[a]: Searched studies published between January 1st 2009 and October 8th 2021.

[b]: Searched studies published between October 11th 2021 and August 10th 2022.

Table S2. Ten criteria for quality assessment of the included quantitative studies

Criteria		Study 1	Study 2	….
**A. General**				
1. Objectives/aims	i. clearly stated;<br>ii. partially/poorly stated;<br>iii. not stated			
	Explain			
	Score			
**B. Methodology and Data Collection**				
2. Population	i.The eligibility criteria is clear, population is clearly defined;<br>ii.The eligibility is not clear, polulation is not clearly defined.			
	Explain			
	Score			
3. Representativeness and Recruitment of the Population	i.Truly representative of the average in the target population (all subjects or random sampling);<br>ii. Somewhat representative of the average in the target population (non-random sampling);<br>iii. Selected group of users; iv. No description of the sampling strategy or unclear			
	Explain			
	Score			
4. Sample Size	i. All subjects in the target population (n ⩾100) or explanation regarding sample size used (regardless of n);<br>ii. No explanation regarding sample size used, unsatisfactory size (n <100) or unclear			
	Explain			
	Score			
5. non-Respondents	i. Comparability between respondent and non-respondent characteristics are established, and the response rate is satisfactory (⩾ 70%);<br>ii.The response rate is unsatisfactory (< 70%), or the comparability between respondents and non-respondents is unsatisfactory (< 70%);<br>iii. No description of the response rate or the characteristics of the responders and the non-responders is unclear			
	Explain			
	Score			
6. Data Collection Tool Used	i.Description of tool (e.g. questionnaire) and development or source thereof used to assess the outcome(s) is/are defined;<br>ii.Description of tool (without development or source) used to assess the outcome(s) is/are clearly defined;<br>iii.Description of tool and/or development thereof used to assess the outcome(s) is poorly, unclearly or not defined.			
	Explain			

Criteria		Study 1	Study 2	….
	Score			
7. Outcome-Related Question(s) and/or Statement(s)	The question(s) and/or statement(s) pertaining to the outcome is/are:<br>i.stated explicitly, clearly, are unambiguous and is/are not phrased with bias towards any particular view;<br>ii.not stated explicitly, clearly or they are ambiguous or is/are phrased with bias towards any particular view			
	Explain			
	Score			
8. Ethical Considerations	i.Informed consent was obtained from participants and ethical approval of study was obtained (if applicable);<br>ii.Unclear/not reported if informed consent was obtained from participants or unclear/not reported if ethical approval was obtained (if applicable);<br>iii.Unclear/not reported if informed consent was obtained from participants AND unclear/not reported if ethical approval of study was obtained (if applicable)			
	Explain			
	Score			
**C. Data Presentation and Analysis**				
9. Clarity	i.Data is presented with utmost clarity including numerators, denominators and missing data;<br>ii.At least some data is unclear requiring guesswork or leaving questions unanswered			
	Explain			
	Score			
10. Consistency	i.There is consistency between the research question, data available and reporting of data;<br>ii.At least some inconsistencies exist between the research question, data available and reporting of the data			
	Explain			
	Score			
**Overall quality assessment**				
High, Medium, Low				
Explain				
Score				

Table S3. Patient costs calculated by observations

Cost category	Total Medical costs			Inpatient costs	Outpatient costs	Non-medical costs	Direct costs	Indirect costs	Overall costs
**Annual cost**	15 studies (21 observations)	12 studies[a] (18 observations)	3 studies[b] (3 observations)	12 studies (33 observations)	5 studies (5 observations)	8 studies (8 observations)	7 studies (7 observations)	3 studies (7 observations)	2 studies (2 observations)
Unweighted average of mean costs (SD, range)	5138.91 [3410.38] [1801.88, 14683.58]	4529.76 [3177.17] [1801.88, 14683.58]	8793.80 [1177.8] [7420.85, 10297.02]	3145.52[1431.99] [1369.46, 8647.46]	625.52 [305.79] [114.55, 865.35]	1486.17 [1425.17] [687.84, 4977.83]	7460.69 [3201.78] [4903.09, 12398.68]	1893.52 [3573.36] [380.33, 9978.13]	14305.74 [11414.23] [6234.66, 22376.81]
Median (IQR) of mean costs	4105.94 [2365.39, 5619.88]	3947.25 [2353.63, 5257.95]	8663.51 [8042.18, 9480.27]	2843.23 [2376.00, 3642.42]	772.07 [573.39, 802.24]	1005.14 [887.14, 1219.58]	6422.39 [5147.958, 11692.84]	464.61 [401.25, 1086.70]	22376.81 [6234.66, 22376.81]
**Annual loss of income** (mean**):** Xiao 2022, advanced gastroesophageal adenocarcinoma	--			--	--	--	--	38892.58	--
**Monthly cost** (mean)**:** Leng 2019, cancer patients at the end-of-life	2316.87			--	--	--	--	--	--
**Costs incurred during the first three-phase treatment** (mean)**:** Ren 2019, children with lymphoblastic leukemia	--			--	--	8247.51	--	11182.34	--
**Cost during the illness** (mean)**:** Yun 2019, Multi-cancer	intestinal: 6729.51; lung: 6594.22;			--	--	--	--	--	--

Cost category	Total Medical costs			Inpatient costs	Outpatient costs	Non-medical costs	Direct costs	Indirect costs	Overall costs
	breast: 5227.89; stomach: 6417.17								
**Cost in 23-58 months:** Zhou 2020, retinoblastoma	18026.17			--	--	14053.76	32079.92	15498.14	47578.06
**Cost in the five years diagnosis:** Zhang 2017, lung cancer	36033.53			18589.93	1631.86	2279.80	38313.33	959.39	39272.72

[a]: These studies reported annual medical costs including inpatient and outpatient costs.

[b]: These studies reported annual medical costs including inpatient, and outpatient costs as well as the cost of self-purchasing drugs from pharmacies.

Table S4. Annual medical costs by subgroups from a multi-center patient survey (four studies), mean US$ (n)

Study	Huang, 2017	Liao,2018	Lei, 2020	Zhang, 2020	Unweighted average cost [SD, range]	Median [IQR]
**Cancer**	colorectal cancer	breast cancer	liver cancer	stomach cancer		
**Total**	5397.75 (2356)	4105.94 (2746)	4056.78 (2223)	5524.95 (2401)	4771.35 [691.68] [4056.78, 5524.95]	4751.84 [4093.65, 5429.55]
**Sex**						
Male	5269.93 (1345)	--	4098.26 (1760)	5537.24 (1677)	4968.48 [624.94] [4098.26, 5537.24]	5269.93 [4684.10, 5403.59]
Female	5567.83 (1011)	--	3896.27 (463)	5496.70 (724)	4986.93 [771.76] [3896.27, 5567.83]	5496.70 [4696.48, 5532.26]
**Age**						
<45	6091.26 (361)	4392.20 (860)	4968.24 (362)	5506.52 (310)	5239.55 [630.14] [4392.20, 6019.26]	5237.38 [4824.23, 5652.71]
45-54	5697.24 (542)	4107.16 (1044)	4319.19 (633)	5891.07 (513)	5003.67 [796.99] [4107.16, 5891.07]	5008.22 [4266.19, 5745.70]
55-64	5352.88 (787)	3873.73 (654)	3849.41 (745)	5534.78 (902)	4652.70 [793.79] [3849.41, 5534.78]	4613.31 [3867.65, 5398.36]
>=65	4830.85 (666)	3604.67 (188)	3328.58 (483)	5239.92 (676)	4251.01 [803.55] [3328.58, 5239.92]	4217.76 [3535.65, 4933.12]
**Clinical stage**						
I	4438.44 (328)	3566.59 (546)	3777.62 (299)	5414.38 (420)	4299.26 [719.68] [3566.59, 5414.38]	4108.03 [3724.87, 4682.42]
II	5306.82 (630)	4018.71 (1236)	3948.31 (493)	5333.29 (347)	4651.78 [668.80] [3948.31, 5333.29]	4662.76 [4001.11, 5313.44]
III	5490.87 (815)	4508.91 (603)	4034.45 (946)	5760.84 (661)	4948.77 [704.06] [4034.45, 5760.84]	4999.89 [4390.30, 5558.36]
IV	5921.37 (559)	5227.64 (285)	4003.15 (379)	5430.35 (933)	5145.63 [706.19] [4003.15, 5921.37]	5328.99 [4921.51, 5553.11]
**Insurance**						
UEBMI	4333.35 (916)	3462.16 (1052)	3379.42 (852)	4298.82 (803)	3868.44 [448.77] [3379.42, 4333.35]	3880.49 [3441.47, 4307.46]
URBMI	4909.42 (446)	3193.10 (518)	3747.32 (400)	4833.26 (404)	4170.77 [727.95] [3193.10, 4909.42]	4290.29 [3608.76, 4852.30]
NCMS	6504.81 (897)	5001.58 (1027)	4713.41 (890)	6545.91 (1136)	5691.43 [840.26] [4713.41, 6545.91]	5753.19 [4929.53, 6515.09]
Commercial insurance	7938.30 (22)	5250.98 (46)	3786.80 (19)	7328.52 (14)	6076.15 [1655.15] [3786.80, 7938.30]	6289.75 [4884.93, 7480.96]
No health insurance	8358.03 (49)	6899.74 (71)	7468.31 (40)	8702.08 (30)	7857.04 [712.85] [6899.74, 8702.08]	7913.17 [7326.17, 8444.04]

Table S5. Non-medical costs for cancer treatment from 12 patient surveys

Study	Participants	Cancer patients (n)	Year	Duration of costs	Definition in the study	Mean Cost (US$)
Huang,2012	lung cancer	402	2011	annual	Costs of transportation, lodging, extra nutrition products for patients, and fees for care workers.	4977.83
Li,2016	lung cancer	218	2014	annual	Costs of transportation, extra nutrition products for patients, and fees for care workers.	927.97
Huang,2017	colorectal cancer	2356	2012-2014	annual	Costs of meals, additional nutrition, transportation, accommodation, fees for care workers, and other expenditures.	1024.64
Liao,2018	breast cancer	2746	2012-2014	annual	Costs of transportation, accommodation, meals and nutritional products, and fees for care workers.	1043.35
Lei,2020	liver caner	2223	2012-2014	annual	Costs of meals, accommodation, transportation and nutritional products, and fees for care workers.	846.31
Sui,2020	pediatric leukemia	242	2018	annual	Costs of transportation, accommodation, and nutritional products.	1395.82
Zhang,2020	Stomach cancer	2401	2012-2014	annual	Costs of meals, accommodation, transportation and nutrition, and fees for care workers.	985.63
Chen,2020	esophageal cancer	184	2019	annual	Costs of transportation, accommodation, meals, nutrition, and other expenditures.	687.84
Che,2016	liver cancer	131	2013	annual	Costs of transportation, nutrition supplements, and nursing worker fee.	106.10
Ren,2019	children with lymphoblastic leukemia	161	2010	costs incurred during the first three-phase treatment	Costs of accommodation, transportation, meals and nutrition, fees for hygiene cleaning products and auxiliary treatment, fees for gifts and treats.	8247.51
Zhou,2020	retinoblastoma	50	2015-2017	during the illness (23-58 months)	Costs of transportation, accommodation, meals, and nutrition.	14053.76
Zhang,2017	lung cancer	195	2014	five years	Costs of transportation, accommodations, extra nutrition, and fees for care workers.	2279.80

Table S6. Medical and non-medical costs from seven studies, mean and percentage of total annual direct costs

Study	Type of cancer	Cancer patients (n)	Year	Location	Duration of costs	Medical costs		Non-medical costs		Direct costs
						US$	%	US$	%	US$
Huang,2012[a]	lung cancer	402	2011	East	annual	7420.85	59.85%	4977.83	40.15%	12398.68
Huang,2017	colorectal cancer	2356	2012-2014	Multi-center	annual[b]	5397.75	84.05%	1024.64	15.95%	6422.39
Liao,2018	breast cancer	2746	2012-2014	Multi-center	annual	4105.94	79.74%	1043.34	20.26%	5149.28
Lei,2020	liver caner	2223	2012-2014	Multi-center	annual[b]	4056.78	82.74%	846.31	17.26%	4903.09
Zhang,2020	stomach cancer	2401	2012-2014	Multi-center	annual[b]	10297.02	88.06%	1395.82	11.94%	11692.84
Sui,2020 [a]	pediatric leukemia	242	2018	East	annual	5524.95	84.86%	985.63	15.14%	6510.59
Chen,2020	esophageal cancer	184	2019	Central	annual	4460.12	86.64%	687.84	13.36%	5147.96

[a]: In these studies, medical costs included inpatient and outpatient costs and fees for purchasing drugs from pharmacies.

[b]:In these studies, costs incurred during 2 months before and 10 months after diagnosis

Table S7. Indirect costs for cancer treatment from seven studies

Study	Participants	Cancer patients (n)	Year	Duration of costs	Data source	Definition in the study	Mean cost (US$)
Huang,2012[a]	lung cancer	402	2011	annual	survey	Income loss of both patients and their family due to illness and financial loss due to seeking non-medical support for the illness.	9978.13
Zhao,2016	cancers	318	2014	annual	survey	No definition	490.31
Chen,2020[a]	esophageal cancer	184	2019	annual	survey	Cost of productivity loss of patients and their family caregivers due to illness.	1086.70
Xiao,2022	advanced gastroesophageal adenocarcinoma	66	2019	annual	survey	Income loss of patients due to illness.	38892.58
Ren,2019	children with lymphoblastic leukemia	161	2010	costs incurred on the first three-phase treatment	survey	Costs of productivity loss of children's parents due to taking care of ill kids (human capital approach).	11182.35
Zhang,2017[a]	lung cancer	195	2014	five years	survey	Income loss of both patients and their family caregivers resulting from outpatient visits and hospitalization (human capital approach)	959.39
Zhou,2020[#]	retinoblastoma	50	2015-2017	during the illness（23-58 months)	survey and medical records	Income loss caused by the loss of working hours of the accompanying parents.	15498.14

[a]: These studies reported both direct cost and indirect costs.

Table S8. Annual direct cost as a percentage of annual household income from six studies and meta-proportion of five studies, %

Study	Participants	Cancer patients (n)	Year	Duration of costs	Annual direct costs (US$)	Annual household income (US$)	Ratio
Huang,2012[a]	lung cancer	402	2011	annual	12398.68	13550.73	91.50%
Huang,2017	colorectal cancer	2356	2012-2014	annual[b]	6422.387	10013.99	64.13%
Liao,2018	breast cancer	2746	2012-2014	annual	5149.28	10990.09	46.85%
Lei,2020	liver caner	2223	2012-2014	annual[b]	4903.091	11259.7	43.55%
Zhang,2020	Stomach cancer	2401	2012-2014	annual[b]	6510.585	9481.498	68.67%
Chen,2020[c]	esophageal cancer	184	2019	annual	5147.957	3454.453	149.02%
Random Pooled ES (95%CI) of the proportion of annual direct cost to annual household income from five studies							64% [44%, 82%]

[a]:In these studies, medical costs included inpatient and outpatient costs and fees for purchasing drugs from pharmacies.

[b]:In these studies, costs incurred during 2 months before and 10 months after diagnosis.

[c]: This study was not included in the meta-proportion analysis.

Table S9. Catastrophic health expenditure (CHE) incurred by cancer care from nine studies

Study	Participants	Cancer patients (n)	Year	Location	Definition of CHE	CHE rates
Zhao,2021	cancer patients	2011:234<br>2015:368	2011, 2015	National level	Annual medical cost more than 40% households' non-food expenditures	2011: 25.12%<br>2015: 27.15%
Sun,2021	breast cancer	639	2015-2016	multi-center		66.82%
Sun,2021 [a]	lung cancer	2565	2015-2016	multi-center		78.1%
Huang,2021[a]	cancer	332	2015-2016	Guangxi		61.14%
Sui,2020	pediatric leukemia	242	2018	Heilongjiang		43.4%
Che,2016	liver cancer	131	2013	Yunnan	Annual direct cost (including medical cost and non-medical cost) more than 40% annual households' income	49.6%
Chen,2018	lung cancer	227	2016	Shanghai		72.70%
Leng,2019	cancer patients at the end-of-life	792	2013-2016	multi-center	Monthly direct cost (including medical cost and non-medical cost) more than 40% monthly households' income	Rural: 96.1%<br>Urban: 94.3%
Zhao,2016	cancer	318	2014	Anhui	Annual inpatient cost more than 40% of annual household income	20.6%

[a] In these studies, costs incurred during 2 months before and 10 months after diagnosis.

Zhao, 2022: Data from a population-based survey, only approximately half of the cancer patients utilized treatment.

Chen,2018: Cost-income ratio was reported by the participants.
